# Vaccination by Private Practitioners

**Published:** 1898-04-09

**Authors:** 


### Vaccination by Private Practitioners.

The *Star* accuses us of stigmatising doctors "as committing fraud wholesale for the sake of a paltry fee," and much else to the same effect, because we have said, in regard to private vaccination, that "the public, the great employer, has paid for what it wanted, namely, just as much 'vaccination' as would enable the practitioner to sign the certificate." We need hardly say that such an accusation is absolutely untrue, and that such a charge could only be made by one ignorant of the bearing of the facts we named. We made no suggestion of fraud or wrong doing, but merely pointed out the well-known reasons for the equally well-known inferiority of private vaccination. We do not, however, complain of the *Star*, because when papers take up the anti-vaccination crusade they must, we suppose, adopt anti-vaccinist methods; but we certainly are surprised that, just for the sake of indulging in an ill-mannered snarl at The Hospital, the *Medical Press and Circular* should join in such an outcry; for if its editors know anything at all about medical work, they must be aware that every word we wrote is absolutely true.

Everyone knows how largely "one mark" vaccination prevails, and that there is no suggestion of fraud in saying that in deference to the wishes of their patients private practitioners make less than the standard four insertions. The Act gives no definition of what it means by successful vaccination, and when a single-mark vaccination is returned as "successful," it is the Act which is to blame. What of course we could not expect the *Star* to know, but what the *Medical Press* ought to have known, is that the Local Government Board asks from the public vaccinators a much higher type of vaccination than is required by the Act, and that the reason why private vaccination is inferior to public vaccination, a point clearly brought out before the Vaccination Commission, is that the private practitioner is employed to vaccinate in such a manner as will satisfy the Act, while the public vaccinator is engaged to do such vaccination as will satisfy the Local Government Board. The doing of a statutory vaccination, however, is no fraud, and it is perfectly well known that for a long time even the medical officer to the small-pox ships of the Metropolitan Asylums Board, in re-vaccinating the persons employed there, only made one mark. Under these circumstances, what are we to think of the *Medical Press* when it says: "There are, doubtless, a few unscrupulous practitioners who are ready to pander to their patients' prejudices in respect of vaccination, just as there are a few who are willing to procure abortion for a sufficient inducement, but the immense majority of practitioners would shrink from the one as from the other."

Such an exaggerated statement as this shows miserable ignorance of the whole subject. If the editor of the *Medical Press* would refer to Allbutt's "System of Medicine" he would find that "there are many medical men" (not "a few unscrupulous practitioners") who are ready to make "one mark;" if he will refer to the minutes of evidence before the Vaccination Commission he will find it repeatedly stated that private vaccination is inferior to public vaccination; he will also find Sir Richard Thorne Thorne saying that "it is a pretty well-known fact that the custom of vaccinating in one place, for example, instead of four has been very habitual in some parts of London," as well as in other districts; so largely, indeed, as to afford some explanation of the excessive incidence of small-pox upon the young in those districts.

We have said nothing that is not perfectly well known in medical circles, and as for our suggestion that if vaccination of a better standard is wanted, it should be paid for, there is surely nothing derogatory to the honour of the profession in that. We would point out that this is exactly what is suggested in the Final Report of the Vaccination Commission, which says, "Safeguards might be provided against inefficient vaccination. The fee should only be allowed in cases where the certificate showed that the child had been vaccinated in accordance with the rules prescribed by the Local Government Board." The *British Medical Journal* goes so far as to say that a proper standard of vaccination "cannot be secured except under a system of inspection and control by a central authority." But the *Medical Press and Circular* knows none of these things, and in face of the well-recognised fact that "a deplorably large proportion of the nominally vaccinated to-day have been most inefficiently vaccinated, and are consequently, in many cases, almost unprotected against an attack of small-pox" ("A System of Medicine"), chooses to say that inefficient vaccination is only done by a few unscrupulous practitioners who pander to their patients' prejudices, and goes so far as to compare those who only produce enough work to enable them to sign the legal certificate to men who "are willing to procure abortion for a sufficient inducement."

All this is pernicious nonsense. We shall never get good private vaccination until we recognise the reason for its present inferiority.

